# Michigan’s Ways

**Published:** 1900-05

**Authors:** 


### MICHIGAN'S WAYS.

Braxy in sheep finds its first location in this State, according to the officials of the Bureau of Animal Industry, though several of Michigan's noted bacteriologists have designated the disease causing such severe and sudden losses in sections of that State as anthrax.

A revised decision of the Bureau of Animal Industry is announced as forthcoming from investigations of a recent serious outbreak of disease among Michigan's sheep. That it is anthrax, as claimed by some of the State investigators, is beyond question.

It was rather a unique situation to see those who had drafted and fathered the veterinary law seeking counsel to save them from the requirements the law provided. An examination as contemplated by the State Board of Veterinary Medical Examiners on the lines proposed might have left some of Michigan's veterinarians without a means of livelihood. Strange things happen these days.

The effort to have Governor Pingree recall the appointment of Dr. George Hare, of Allegan, Mich., as a member of the State Board of Examiners proved unsuccessful; Dr. Hare's claim to have practised three years before graduation was considered sufficient to establish his eligibility.

According to Michigan's State Board, a six-months' college course is to be considered as complied with by sessions of a few days in excess of five months. A better rendering would save much future trouble on this score.

The decision of the State Board of Veterinary Medical Examiners to admit to registration all graduates of the Ontario Veterinary College relieved the minds of many and prevented a bitter fight all over the State over the law regulating the practice of veterinary science.

Will the Michigan Association admit graduates of the only remaining veterinary school within their borders to membership?

Do the Michigan veterinarians threaten to boycott the JOURNAL for telling the truth; if not, what then?

The charges and counter-charges indulged in by Michigan's representatives of the profession as to who are responsible for the inadequacy of the State veterinary law's shortcomings seems past finding out. To be on varying sides of the measure most to be desired seems to be the record of many of Michigan's veterinarians at the several sessions of the Legislature when this legislation was foremost.

